# Effect of Global Regulators RpoS and Cyclic-AMP/CRP on the Catabolome and Transcriptome of *Escherichia coli* K12 during Carbon- and Energy-Limited Growth

**DOI:** 10.1371/journal.pone.0133793

**Published:** 2015-07-23

**Authors:** Alessandro G. Franchini, Julian Ihssen, Thomas Egli

**Affiliations:** 1 Eawag, Swiss Federal Institute of Aquatic Science and Technology, Dübendorf, Switzerland; 2 Institute of Biogeochemistry and Pollutant Dynamics, ETH Zurich, Zurich, Switzerland; University of Houston, UNITED STATES

## Abstract

For heterotrophic microbes, limited availability of carbon and energy sources is one of the major nutritional factors restricting the rate of growth in most ecosystems. Physiological adaptation to this hunger state requires metabolic versatility which usually involves expression of a wide range of different catabolic pathways and of high-affinity carbon transporters; together, this allows for simultaneous utilization of mixtures of carbonaceous compounds at low concentrations. In *Escherichia coli* the stationary phase sigma factor RpoS and the signal molecule cAMP are the major players in the regulation of transcription under such conditions; however, their interaction is still not fully understood. Therefore, during growth of *E*. *coli* in carbon-limited chemostat culture at different dilution rates, the transcriptomes, expression of periplasmic proteins and catabolomes of strains lacking one of these global regulators, either *rpoS* or adenylate cyclase (*cya*), were compared to those of the wild-type strain. The inability to synthesize cAMP exerted a strong negative influence on the expression of alternative carbon source uptake and degradation systems. In contrast, absence of RpoS increased the transcription of genes belonging to high-affinity uptake systems and central metabolism, presumably due to reduced competition of σ^D^ with σ^S^. Phenotypical analysis confirmed this observation: The ability to respire alternative carbon substrates and to express periplasmic high-affinity binding proteins was eliminated in *cya* and *crp* mutants, while these properties were not affected in the *rpoS* mutant. As expected, transcription of numerous stress defence genes was negatively affected by the *rpoS* knock-out mutation. Interestingly, several genes of the RpoS stress response regulon were also down-regulated in the cAMP-negative strain indicating a coordinated global regulation. The results demonstrate that cAMP is crucial for catabolic flexibility during slow, carbon-limited growth, whereas RpoS is primarily involved in the regulation of stress response systems necessary for the survival of this bacterium under hunger conditions.

## Introduction

As a rule, two major factors control heterotrophic bacterial growth in most ecosystems: On the one hand it is temperature, as it controls the rate of biochemical processes (and in most ecosystems temperatures are low); on the other hand, availability of assimilable carbon and energy sources is severely restricted, as most of the potentially utilizable carbon compounds are present in polymeric forms and are not directly accessible [1,2]. This latter situation, usually referred to as oligotrophy, starvation, or (technically) carbon and energy limited conditions, has also been described physiologically as the “hunger status” [3]. Availability of organic nutrients (particularly of assimilable organic carbon, AOC) is the result of the usually slow rate of AOC generation from, e.g, decaying plant material and its consumption by the competing and interacting microbial cells present in a given environment. In addition, microbial cells are exposed very directly to potentially harmful changes in temperature, light and other physico-chemical parameters. Hence, they have to be prepared for such challenges, and when necessary, also to be able to rapidly react to environmental stresses. Microbial cells are known to be able to adjust their cellular composition to environmental needs [4] and they possess fine-tuned global regulatory mechanisms that allow coordinated gene expression in complex regulatory networks resulting from the interaction of different global regulators [5–7].

As carbon and energy availability is a major factor determining the survival and proliferation of microbes in ecosystems, a rapid response to fluctuations in nutrient concentrations is an important fitness determinant [8–9]. An increased catabolic versatility and flexibility, allowing the simultaneous uptake and utilization of multiple organic nutrients and a rapid adjustment of uptake spectrum and fluxes according to availability in the immediate environment, is—in contrast to carbon (glucose) catabolite repression—a useful physiological strategy under hunger conditions [8,10–12]. Furthermore, cells respond to hunger by expressing a variety of high-affinity uptake systems involved in carbon and energy substrate transport [8, 13–18].

Concerning the numerous adverse physical and chemical challenges a cell might face, a broadly expressed stress response is an essential requirement for successful survival. Therefore, slowly growing bacterial cells also depend on the induction of general stress response mechanisms [19]. Even more important here is to be prepared for all possible situations as there are neither sufficient resources to express stress response and defence systems in times of need, nor is there probably enough time to react [11]. Two global regulators are known to control nutrient scavenging abilities and stress response systems in the model bacterium *E*. *coli*, namely cAMP-CRP and the alternative (stress response) sigma factor RpoS. Under glucose-limited conditions intracellular concentrations of cAMP are increased and a (strain specific) correlation between specific growth rate and cAMP in glucose-limited continuous culture was found [20–23]. A similar increase was observed for RpoS levels during the transition from exponential growth phase to stationary phase in complex medium, where during slow growth RpoS reached 30–50% of the levels of the housekeeping sigma factor RpoD [24]. A direct correlation between increasing RpoS-levels and decreasing specific growth rates was also reported for exponentially growing batch cultures and carbon-limited continuous cultures of *E*. *coli* [19,25], whereas levels of RpoD remained constant [25]. When investigating and comparing gene expression and global physiology of microbes under different growth conditions it is necessary to control the cell’s environment as well as possible [4,26]. Of particular importance with respect to overall cellular composition and global response are two parameters, namely the type of the growth-limiting nutrient (whether C, N, P etc.), and the specific growth rate [4,17,27–29]. With the exception of the phase of genuinely exponential and nutritionally unrestricted growth in batch culture [17], steady-state continuous (or chemostat) cultivation is the only method that allows a precise control of the specific growth rate by a selected limiting nutrient of bacterial cells, and thus of their physiological state [4,26,30]. Regarding slow, nutrient-limited growth, chemostat culture seems to be the only well reproducible experimental approach [18,31]. Although numerous studies addressed the effect of knock-out mutations of global regulatory genes on the transcriptome of *E*. *coli* in batch culture, similar experiments under constant, carbon- and energy-limited growth conditions in chemostats are largely missing. Transcriptome studies of global regulatory mutant strains in batch culture were performed for RpoS [32–37], cAMP-CRP [38,39], Lrp [40,41], and Crl [42]. Already in 2002 a cross-talk between cAMP and RpoS was proposed [43]. Later many genes, known to be RpoS-dependent, were reported to be also regulated by either cAMP [38], or to have a cAMP-CRP binding site within the promoter region in transcriptome/genome studies [37]. However, results are far from being consistent; several operons seem to be also under the control of additional global regulators, indicating a complex mechanism governing gene expression.

In an attempt to disentangle the effects of the cAMP-CRP and RpoS global regulators and their roles for physiological adaptation to (environmentally relevant) slow growth exerted by limited availability of carbon and energy sources, we compared the transcriptome and global physiological state of wild-type versus Δ*rpoS* or Δ*cya E*. *coli* strains during cultivation in glucose- and LB-limited chemostat culture.

## Material and Methods

### Bacterial strains, growth medium and growth conditions

Wild-type *E*. *coli* MG1655 and *rpoS* knock-out strains used in this study have been described elsewhere [18,31]. The Δ*cya* and Δ*crp* strains are also derivatives of *E*. *coli* MG1655 and were obtained from A. C. Matin and M. Cashel [44]. The genotype of all strains is given in Table 1. Glucose-limited mineral medium and carbon-limited modified LB medium for batch and chemostat cultivation were described previously [15,25]. The Δ*cya* strain exhibited a strongly reduced growth yield in modified LB medium, therefore, for chemostat cultivation tryptone and yeast extract concentrations were increased to 2 g L^-1^ and 1 g L^-1^, respectively, (which corresponds to 20% of the original strength of LB medium). Cultivation conditions were the same as in a previous study [15]. For continuous cultivation small volume glass bioreactors were used. Aeration with a fine pore glass purger and magnetic stirring was sufficient to keep oxygen saturation above 60%. For batch cultivation with modified LB medium, baffled Erlenmeyer flasks (300 ml) and a low liquid volume (50 ml) were used. Flasks were vigorously shaken as electromagnetic stirring was not sufficient for maintaining fully oxic conditions during the transition to stationary phase. To avoid adaptive mutations in the wild-type strain and compensatory mutations in knock-out strains [18], new starter cultures were prepared for each experiment. Stability and purity of chemostat cultures were checked for wild-type and Δ*rpoS* strains as described previously [25] and for Δ*cya* and Δ*crp* strains by confirming in addition the strongly reduced number of substrates which support growth on BIOLOG AN MicroPlates.

**Table 1:** Effect of global regulatory mutations on growth kinetic parameters and specific hydroperoxidase activity of *E*. *coli* in glucose mineral medium cultures at 37°C. The dilution rate in glucose-limited chemostat cultures was set at a value of approximately half the maximum specific growth rate (D = 0.3 h^-1^ for wild-type and Δ*rpoS* strains, D = 0.16 h^-1^ for the Δ*cya* strain and D = 0.14 h^-1^ for the Δ*crp* strain). Values represent the average of replicate measurements (n ≥ 3), standard deviations are given in parentheses.

strain	genotype	μ_max_	K_s_	Yield	HPI+II ^a^	HPII ^b^
		[h^-1^]	[mg L^-1^]	[g _dr. wt._ g _glc._^-1^]	[μmol H_2_O_2_ min^-1^ mg_protein_^-1^]	
MG1655 (wt)	*F* ^*-*^ *λ* ^*-*^ *rph1*	0.63^c^	0.54	0.46^d^	62.9^d^	27.7^d^
		(± 0.01)	(± 0.12)	(± 0.01)	(± 8.1)	(± 3.9)
*ΔrpoS*	MG1655*ΔrpoS*::*Tn10*	0.69	0.29	0.37	67.0^d^	1.1^d^
		(± 0.01)	(± 0.03)	(± 0.06)	(± 3.4)	(± 0.2)
*Δcya*	MG1655*Δcya-854*	0.35	5.7	0.33	51.0	23.3
		(± 0.03)	(± 1.2)	(± 0.04)	(± 16)	(± 4.7)
*Δcrp*	MG1655 *Δcrp-39 rpsL*	0.28	19.6	0.31	n. d.^e^	n. d.^e^
		(± 0.03)	(± 1.8)	(± 0.02)		

^a^ combined activities of heat labile hydroperoxidase I (KatG) and heat stable hydroperoxidase II (KatE)

^b^ heat stable hydroperoxidase II activity (KatE)

^c^ data from [18]

^d^ data from [25]

^e^ not determined

Cultures were first run in the batch mode and growth was followed by determining OD_546_ spectrophotometrically (model Uvikon 930, Kontron, Zurich, Switzerland) at regular intervals. After 4–5 hours the culture mode was changed to continuous at a dilution rate corresponding to half μ_max_ by switching on the medium feed pump. Steady-state with respect to optical density was reached approximately 20 hours after starting the medium feed. Samples for catabolic pathway assays, periplasmic proteins and transcriptome analysis were taken 40 hours after switching to the continuous culture mode, and each experiment was done in triplicate.

### Determination of growth kinetic parameters, enzyme activities and catabolic ability

Maximum specific growth rates (μ_max_, h^-1^) were determined for growth in both mineral glucose and modified LB medium batch cultures at 37°C. To avoid lag phases, pre-warmed medium was inoculated from exponential phase cultures growing in the same medium. Specific growth rates were calculated from the linear part of logarithmic growth curves by least squares regression. The linear part of logarithmic growth curves in modified LB medium ended at an OD_546_ of approximately 0.2 (see [45]), higher optical densities were excluded from the calculation of μ_max,LB_. Growth yield was determined as described in Ihssen and Egli [25]. Steady-state glucose concentrations were determined by ion chromatography [25], the results were used for the calculation of glucose affinities (K_s,Glc_, μg L^-1^) with a modified Monod equation including an s_min_ of 12 μg L^-1^ [46].

Expression of catabolic pathways in carbon-limited chemostat cultures was analyzed with BIOLOG AN MicroPlate respiration assays [15]. Periplasmic proteins were extracted by chloroform shock treatment and analysed on 15% SDS-PAGE gels which were stained with Coomassie Blue [15]. Hydroperoxidase I and II specific activities were determined with a spectrophotometric assay as described previously [25].

### RNA isolation and cDNA synthesis

Total RNA was isolated using standard procedures [47]. The RNA extracted was checked for purity by gel electrophoresis and was quantified by measuring absorbance at 260 nm. CyScribe First-Strand cDNA Labelling Kit (Amersham Bioscience, Little Chalfont, England) was used to reversely transcribe mRNA into fluorescently labelled cDNA, followed by hybridization onto the microarray slide as previously described [13]. Slide microarrays were purchased from MWG-Biotech AG (MWG *E*. *coli* K12 V2 array, Ebersberg, Germany); they contained gene specific oligonucletide probes representing the complete *E*. *coli* K12 genome (4,288 ORF).

### Image data analysis

Microarray slides were scanned with an Affymetrix 428 Array Scanner (High Wycombe, England) and the obtained images were analysed with the Affymetrix Jaguar software version 2.0. Spot intensities and corresponding background signals were further analyzed with the program GeneSpring from Silicon Genetics (Redwood City, USA) as reported elsewhere [13]. After background correction, the mean value of three replicate spots per sample was used for calculation of induction/repression factors of individual genes. Data from the three independent experiments were combined, genes that were differently regulated ≥3 and ≤-3 (t-test, p ≤ 0.2) were defined as being statistically significant. We did not choose a stricter p value because gene expression levels of *Δcya* and *ΔrpoS* strains exhibited a large variation after normalization. Technical replicates on the same slide exhibited a signal variability below 10%, but a much higher variance was observed for gene expression ratios of biological replicates after normalization, in particular for genes with low expression in one of the two compared strains. A multitude of genes exhibited an up- or down-regulation factor between 2 and 3. We considered this as background variability and therefore set the threshold of for significant effects of the studied global regulatory mutations as ≥3 and ≤-3. Validity of this value is backed by results for genes previously reported under the control of cAMP-CRP [38,39]. Although exhibiting a high variability of expression ratios, the mean value was always above 3 when comparing the wild-type and the *Δcya* strain, for example, *ptsG* (4.0 ± 1.4, from 3.0 to 5.6), *mglA* (3.9 ± 2.6, from 2.2 to 6.9), *mglB* (4.9 ± 1.5, from 3.4 to 6.4), *rbsD* (3.6 ± 0.8, from 3.0 to 4.5) and *mdh* (4.1 ± 1.0, from 3.0 to 5.0). The same was observed for genes known to be regulated by RpoS, for example *idnT* (-3.0 ± 0.85, from -2-1 to -3.75), *prpE* (5.9 ± 2.8, from 3.5 to 9.0) and *talA* (8.6 ± 7.3, from 3.2 to 16.9) all surpassed the threshold of the expression ratios when comparing wild type and *ΔrpoS* strains. The complete set of microarray data is available on the Gene Expression Omnibus (GEO) website (http://www.ncbi.nlm.nih.gov/geo/) under the accession number GSE25982.

## Results and Discussion

Growth of heterotrophic microbes in the natural environment is most of the time restricted by the availability of carbon and energy sources [48]. This is also the case for *E*. *coli*, even in the gut periods of real abundance are probably rare and short, followed virtually immediately by limitation. The situation was described earlier as feast and famine existence [49], with famine being the dominating nutritional condition outside its primary habitat [2], because concentrations of easily usable monomeric growth substrates such as sugars, amino acids and organic acids in the environment are usually in the range of 1–100 μg L^-1^ [50]. Even though *E*. *coli* cannot be considered an oligotrophic microorganism, steady-state concentrations of the limiting carbon substrate (glucose) in slow-growing chemostat cultures were 50 μg L^-1^ and lower (D ≤ 0.1 h^-1^), indicating that wild-type *E*. *coli* MG1655 is capable of transporting and metabolizing minute amounts of organic nutrients [31]. Yet more, the ability to survive, grow and even compete with the natural bacterial flora in freshwater was recently confirmed also for *E*. *coli* O157 [51]. The physiological state during carbon-limited growth is fundamentally different from that of cells growing with excess carbon and energy sources; the former is characterized by an up-regulation of a multitude of high-affinity transport systems and catabolic pathways for diverse organic nutrients [13,15,18].

As both, cAMP and RpoS are considered to be the two global regulators responsible for carbon- and energy-limitation physiological phenotype (hunger phenotype), we have investigated the effect of the two regulators under comparable growth conditions in either glucose- or LB-limited chemostat culture.

### cAMP-CRP is strictly required for expression of the hunger phenotype

The extent to which the hunger phenotype depends on the cAMP-CRP global regulator was investigated by comparing the isogenic wild-type strain *E*. *coli* K12 MG1655 to two knock-out mutants, deficient in either adenylate cyclase (*cya*) or in CRP, during growth with a defined mineral glucose medium in carbon- and energy-limited chemostat culture. Transcriptome analysis of the wild-type and the isogenic *Δcya* strain was performed in three replicate glucose-limited chemostat cultures operated at a dilution rate (D) corresponding to approximately half μ_max_,_Glc_, i.e. at extracellular glucose concentrations corresponding to K_s,Glc_; hence, the wild-type strain was grown at a D of 0.3 h^-1^, whereas the *Δcya* strain was cultivated at a D of 0.16 h^-1^ (see Table 1). This choice was made because, first, wild-type *E*. *coli* exhibited the best K_s_ to glucose at a dilution rate of 0.3 h^-1^ [31] and, second, because this dilution rate equivalent to half μ_max_ was used in earlier proteome [18], catabolome [15] and transcriptome analyses [13] performed with wild-type *E*. *coli* K12 MG1655. Due to the fact that μ_max_,_Glc_ of the *Δcya* strain in mineral medium was reduced by 50% (Table 1), which is in agreement with a previous study [52], a D of 0.16 h^-1^ was chosen for cultivating this mutant (0.14 h^-1^ for the *Δcrp* strain), in order to obtain stable growth under glucose-limited continuous culture conditions. Similar levels of KatE (belonging to the RpoS regulon) activity were found in both wild-type and *Δcya E*. *coli* strain, compared to the low levels present in the *ΔrpoS* mutant (Table 1), which suggests minor secondary effects on RpoS levels in the *Δcya* strain resulting from the reduced μ_max_,_Glc_. The transcriptome results for the wild-type and *Δcya* strain cultivated under glucose-limited chemostat conditions are shown in Table 2 and S1 Table. Applying a rather strict cut-off factor of 3 for defining a significant difference in relative gene expression, 192 genes were affected by the adenylate cyclase knock-out mutation (Table 2 and S1 Table). The majority of these genes (152) was down-regulated, which is in agreement with the role of cAMP-CRP as a transcription activator. A large fraction of the *Δcya* affected genes (51) were reported to possess one or more cAMP-CRP binding sites in their promoter region [53,54] and 44 of them were previously found to be down-regulated in *crp* mutant strains [38,39]. This observation is in agreement with previous studies reporting that cAMP-CRP regulates more than 400 genes, making it the broadest global regulator after the housekeeping sigma factor RpoD [6].

**Table 2:** Genes coding for proteins involved in transport and metabolism of carbon substrate with significant up- or down-regulation in Δ*rpoS* and Δ*cya* strains (difference in average signal intensity compared to wild-type *E*. *coli* K12 ≥ 3 or ≤ -3, p-value ≤ 0.2) in glucose-limited continuous culture cultivated at D = 0.3 h^-1^.

Gene	b no.	Gene product	Ratio cAMP	Ratio RpoS
**Transport**				
*nhaA*	b0019	Na+/H antiporter, pH dependent	4.1	
*yajR*	b0427	putative transport protein	-3.3**	
*nmpC* ^b^ ^,^ ^e^ ^,^ ^f^ ^,^ ^g^	b0553	outer membrane porin protein, locus of qsr prophage	6.3	-3.2*
*cusB*	b0574	outer membrane transport protein involved in copper tolerance	-3.1*	
*fepE*	b0587	ferric enterobactin (enterochelin) transport	-3.0**	
*cstA* ^f^ ^,^ ^i^	b0598	carbon starvation protein	7.6	
*ybeJ*	b0655	glutamate/aspartate periplasmic binding transport protein	5.4	
*artI* ^g^	b0863	arginine 3rd transport system periplasmic binding protein	4.1	
*ycaD*	b0898	putative transport protein	4.4	
*ompF* ^f^ ^,^ ^i^	b0929	outer membrane protein 1a	27.4*	
*ompA* ^f^ ^,^ ^g^	b0957	outer membrane protein 3a	4.0	
*ycdG*	b1006	putative transport protein	3.6	
*ptsG* ^c^ ^,^ ^f^ ^,^ ^g^ ^,^ ^i^	b1101	PTS system, glucose-specific IIBC component	4.0	
*nhaB*	b1186	Na+/H+ antiporter, pH independent	-4.5**	-5.4**
*ydcS* ^e^	b1440	putative ABC transporter periplasmic binding protein	8.9	9.8
*ydcU*	b1442	putative transport system permease protein	11.9	7.3
*xasA* ^e^ ^,^ ^h^	b1492	acid sensitivity protein, putative transporter		34.1*
*ego*	b1513	putative ATP-binding component of a transport system	15.2	14.3
*manX* ^c^ ^,^ ^f^ ^,^ ^g^ ^,^ ^i^	b1817	PTS enzyme IIAB, mannose-specific	8.0	
*manY* ^c^ ^,^ ^f^ ^,^ ^g^	b1818	PTS enzyme IIC, mannose-specific	15.1	
*manZ* ^f^ ^,^ ^g^ ^,^ ^i^	b1819	PTS enzyme IID, mannose-specific	5.8	
*gatC* ^c^ ^,^ ^i^	b2092	PTS system galactitol-specific enzyme IIC	35.3*	
*gatB* ^c^ ^,^ ^i^	b2093	galactitol-specific enzyme IIB of phosphotransferase system	60.6*	
*gatA* ^c^	b2094	galactitol-specific enzyme IIA of phosphotransferase system	55.9*	
*yohC* ^e^	b2135	putative transport protein	10.1	7.8
*mglA* ^c^ ^,^ ^f^ ^,^ ^g^ ^,^ ^i^	b2149	ATP-binding component of methyl-galactoside transport	3.9	
*mglB* ^c^ ^,^ ^f^ ^,^ ^g^ ^,^ ^i^	b2150	galactose-binding transport protein	4.9	
*exbD*	b3005	uptake of enterochelin tonB-dependent uptake of B colicins	-3.0	
*mscL*	b3291	mechanosensitive channel	6.0	
*feoA*	b3408	ferrous iron transport protein A	-4.9**	
*hdeD* ^b^ ^,^ ^e^	b3511	putative transporter protein		11.5
*dppA*	b3544	dipeptide transport protein	11.9	
*rbsD* ^g^ ^,^ ^i^	b3748	D-ribose high-affinity transport system, membrane-associated protein	3.6	
*rbsB* ^g^	b3751	D-ribose periplasmic binding protein	9.4	
*glpF* ^c^ ^,^ ^f^ ^,^ ^i^	b3927	facilitated diffusion of glycerol	9.6	
*yjbB* ^i^	b4020	putative alpha helix protein	-3.6	
*malG* ^f^ ^,^ ^g^ ^,^ ^i^	b4032	part of maltose permease, inner membrane	5.9	
*malF* ^f^ ^,^ ^g^ ^,^ ^i^	b4033	part of maltose permease, periplasmic	6.9	
*malE* ^c^ ^,^ ^f^ ^,^ ^g^ ^,^ ^i^	b4034	periplasmic maltose-binding protein	7.5	
*malK* ^c^ ^,^ ^f^ ^,^ ^g^ ^,^ ^i^	b4035	ATP-binding component of transport system for maltose	4.2	
*lamB* ^c^ ^,^ ^f^ ^,^ ^g^ ^,^ ^i^	b4036	phage lambda receptor protein, maltose high-affinity receptor	8.1	
*malM* ^c^ ^,^ ^f^ ^,^ ^g^ ^,^ ^i^	b4037	periplasmic protein of mal regulon	12.5	
*actP* ^f^	b4067	acetate permease	10.7	
*alsB*	b4088	D-allose-binding periplasmic protein		-3.2**
*cycA*	b4208	transport of D-alanine, D-serine, and glycine	4.0	
*ytfQ*	b4227	putative D-ribose transport protein, ABC superfamily	3.6	
*ytfR*	b4228	putative ATP-binding component of a transport system	5.2	
*idnT* ^f^ ^,^ ^g^	*b4265*	*L-idonate transporter*	-3.3*	-3.0
*sgcX*	*b4305*	*putative PTS transport system*	4.4	
**Carbon & Energy metabolism**				
*acnB* ^b^ ^,^ ^f^	b0118	aconitate hydrase B		-3.0**
*prpB* ^b^ ^,^ ^f^	b0331	putative carboxyphosphoenolpyruvate mutase	17.0	15.8
*prpC* ^f^	b0333	citrate synthase, propionate metabolism	30.8*	17.6
*prpD* ^f^	b0334	2-methyl citrate dehydratase	5.6	7.6
*prpE* ^f^	b0335	putative propionyl-CoA synthetase		5.9
*yajO*	b0419	putative NAD(P)H-dependent xylose reductase	3.7	
*thiJ*	b0424	4-methyl-5(beta-hydroxyethyl)-thiazole monophosphate synthesis		6.1
*gltA* ^c^ ^,^ ^f^ ^,^ ^g^	b0720	citrate synthase	6.9	
*sdhD* ^a^ ^,^ ^b^ ^,^ ^i^	b0722	succinate dehydrogenase, hydrophobic subunit		-3.1
*sdhB* ^f^ ^,^ ^g^ ^,^ ^i^	b0724	succinate dehydrogenase, iron sulfur protein	7.2	
*sucA* ^c^ ^,^ ^g^ ^,^ ^i^	b0726	2-oxoglutarate dehydrogenase (decarboxylase component)	3.7	
*sucC* ^c^ ^,^ ^g^	b0728	succinyl-CoA synthetase, beta subunit	4.4	
*moa*	b0781	molybdopterin biosynthesis, protein A	-6.2**	-7.4**
*dmsB*	b0895	anaerobic dimethyl sulfoxide reductase subunit B	-7.4**	-3.0**
*mgsA*	b0963	methylglyoxal synthase	5.5	
*nark*	b1223	nitrite extrusion protein		4.7
*aldA* ^c^ ^,^ ^f^ ^,^ ^g^	b1415	aldehyde dehydrogenase, NAD-linked	6.5	
*gapC_1* ^b^	b1417	glyceraldehyde 3-phosphate dehydrogenase C, interrupted		5.6
*astA*	b1747	arginine succinyltransferase	7.0	
*gapA* ^g^	b1779	glyceraldehyde-3-phosphate dehydrogenase A	3.5	
*gatD* ^c^ ^,^ ^f^ ^,^ ^i^	b2091	galactitol-1-phosphate dehydrogenase	11.2	
*gatZ* ^c^ ^,^ ^f^	b2095	putative tagatose 6-phosphate kinase 1	54.4	
*gatY* ^f^	b2096	tagatose-bisphosphate aldolase 1	50.5**	
*fbaB* ^b^ ^,^ ^d^ ^,^ ^e^ ^,^ ^h^	b2097	fructose-biphosphate aldolase	4.0	5.6
*menB*	b2262	dihydroxynaphtoic acid synthetase		-3.0*
*nuoH*	b2282	NADH dehydrogenase I chain H	3.7	
*nuoE*	b2285	NADH dehydrogenase I chain E	4.3	
*talA* ^a^ ^,^ ^b^ ^,^ ^d^ ^,^ ^e^ ^,^ ^h^	b2464	transaldolase A		8.6
*eno*	b2779	Enolase	3.6	
*glcB*	b2976	malate synthase G	4.1	
*glcF*	b2978	glycolate oxidase iron-sulfur subunit	7.1	
*glcD*	b2979	glycolate oxidase subunit D	7.0	
*mdh* ^f^ ^,^ ^g^ ^,^ ^i^	b3236	malate dehydrogenase	4.1	
*pckA* ^g^	b3403	phosphoenolpyruvate carboxykinase	8.7	
*atpD* ^g^	b3732	membrane-bound ATP synthase, F1 sector, beta-subunit	4.7	
*atpH* ^g^	b3735	membrane-bound ATP synthase, F1 sector, delta-subunit	4.0	
*atpE* ^g^	b3737	membrane-bound ATP synthase, F0 sector, subunit c	4.6	
*atpB* ^g^	b3738	membrane-bound ATP synthase subunit a	5.3	
*aceB* ^c^ ^,^ ^f^	b4014	malate synthase A	4.1	
*aceA* ^c^ ^,^ ^f^ ^,^ ^i^	b4015	isocitrate lyase	5.1	
*acs* ^f^ ^,^ ^g^ ^,^ ^i^	b4069	acetyl-CoA synthetase	19.5	
*frdD*	b4151	fumarate reductase, anaerobic, membrane anchor polypeptide	4.1	
*frdB* ^b^	b4153	fumarate reductase, anaerobic, iron-sulfur protein subunit	-3.1**	-3.2**

* p-value < 0.1,

** p-value < 0.05

References:

^a^[32]

^b^[33]

^c^[38]

^d^[35]

^e^[36]

^f^[52]

^g^[53]

^h^[37]

^i^[39]

**Fig 1:**
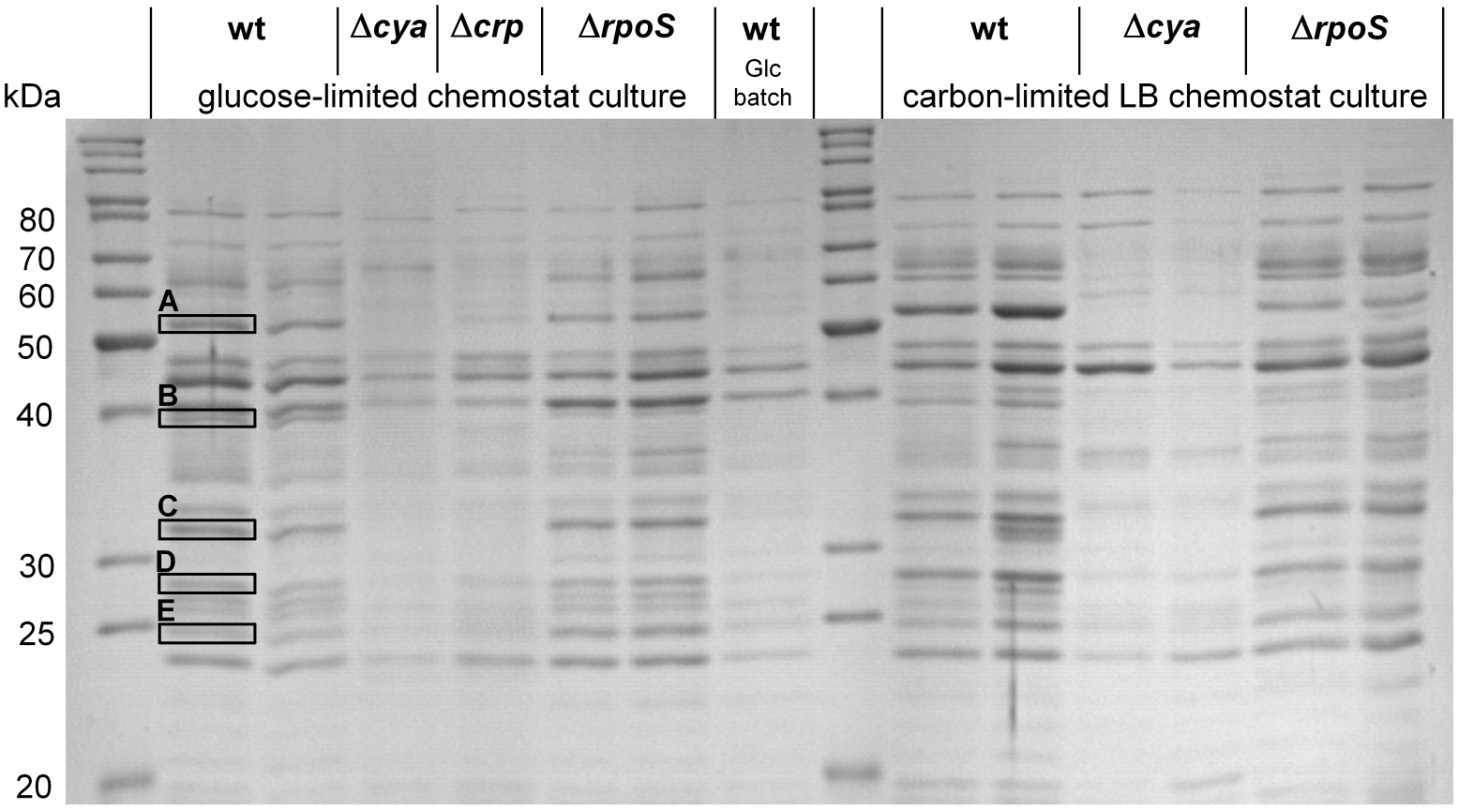
Expression of periplasmic proteins in carbon-limited chemostat cultures of wild-type (wt),Δ*crp*, Δ*cya* and Δ*rpoS E*. *coli* strains. Carbon-limited LB chemostat cultures were set at a dilution rate of 0.3 h^-1^, while the dilution rate of glucose-limited chemostat cultures was operated at approximately half the maximum specific growth rate (D = 0.3 h^-1^ for wild-type and *rpoS* strains, D = 0.16 h^-1^ for the*cya* strain and D = 0.14 h^-1^ for the*crp* strain). The protein bands marked in the figure were identified in a previous study [15] as (A) dipeptide-binding protein DppA, (B) maltose-binding protein MalE, (C) galactose/glucose-binding protein MglB, (D) ribose-binding protein RbsB and (E) glutamine-binding protein GlnH. When two lanes per strain for a similar condition are shown, chloroform shock extracts from two separate chemostat cultures were loaded onto the gel. Loading volumes were normalized to the optical density of the culture samples. Protein size markers are shown in the first and ninth lane from the left.

Most of the genes down-regulated in the Δ*cya* strain belong to operons involed in the uptake of carbon sources (Table 2): glucose (*ptsG*), maltose (*malEFG* and *malK*-*lamB*-*malM*), galactose (*gatABCD* and *mglBA*), ribose (*rbsDB* and *ytfQR*), mannose (*manXYZ*) and acetate (*actP*). Genes for the uptake of amino acids were also affected, e.g., those for glutamate and aspartate (*ybeJ*), arginine (*artI*), alanine, serine and glycine (*cycA*), peptides (*cstA*) and dipeptides (*dppA*). Several outer membrane porins (*nmpC*, *ompF*, *ompA* and *ompC*), a mechanosensitive channel (*mscL*), and an antiporter (*nhaA*) were also down-regulated in the Δ*cya* strain, whereas another antiporter (*nhaB*) was up-regulated. The down-regulation of several genes encoding proteins involved in the transport of carbon sources in the absence of cAMP is in agreement with the results of previous studies performed in batch cultures [38,39]. Among the cAMP-dependent transporter genes found in this study, the *mal* operon, *mgl* operon and *ompF* have previously been shown to be important for high-affinity uptake of glucose under carbon-limited conditions and to be under the transcriptional control of cAMP [18,55–57]. The low expression of genes encoding ABC-type high-affinity uptake systems and the PTS transporter for glucose is in agreement with the 10 to 20-fold poorer affinity (K_s,Glc_) of both Δ*cya* and Δ*crp* strains for glucose as determined from steady-state concentrations in chemostat cultures (Table 1). In contrast, the growth yield for glucose was only marginally affected by these global regulatory mutations, indicating that biosynthetic pathways are not part of the cAMP-CRP and RpoS regulons (Table 1). The transcriptome data and the increased K_s,Glc_ are consistent with the missing over-expression of high-affinity periplasmic binding proteins for carbon substrates in the Δ*cya* and Δ*crp* strains under carbon- and energy-limited growth conditions (Fig 1). Expression patterns for periplasmic proteins in Δ*cya* and Δ*crp* strains were similar, although minor differences existed. Interestingly, such minor differences in transcriptome analyses between Δ*cya* and Δ*crp* strains were also reported for *Vibrio cholerae* [58].

**Fig 2:**
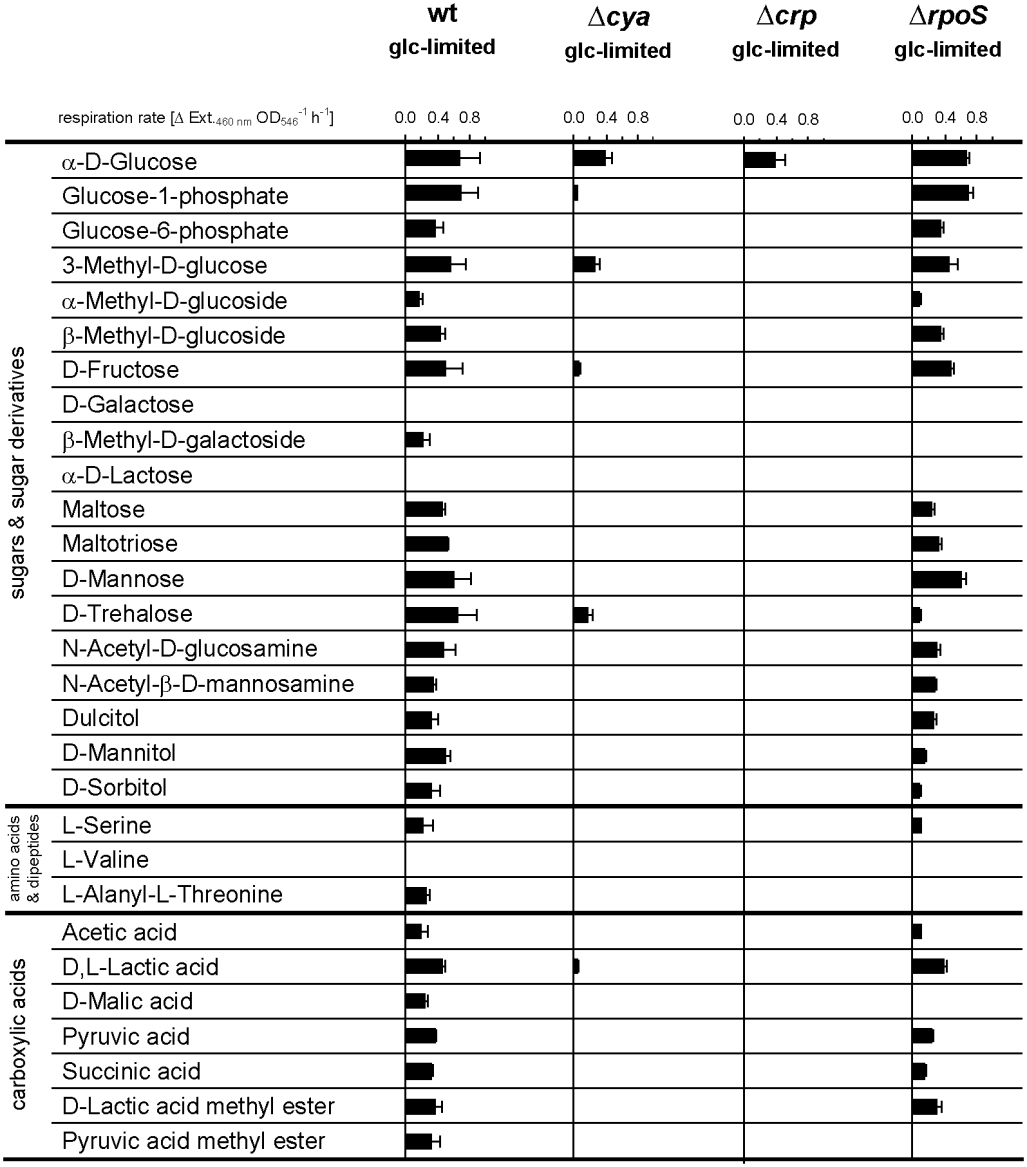
Substrate respiration rates of wild-type, Δ*crp*, Δ*cya* and Δ*rpoS* strains of *E*. *coli* in glucose-limited chemostat cultures. Respiration rates were determined with chloramphenicol-inhibited cells on BIOLOG AN MicroPlates and normalized to OD_546_ of the used cell suspension. Bars give average rates of ≥ 4 BIOLOG plates, for each strain cells were taken from two separate chemostat cultures. Error bars represent standard deviations.

**Fig 3:**
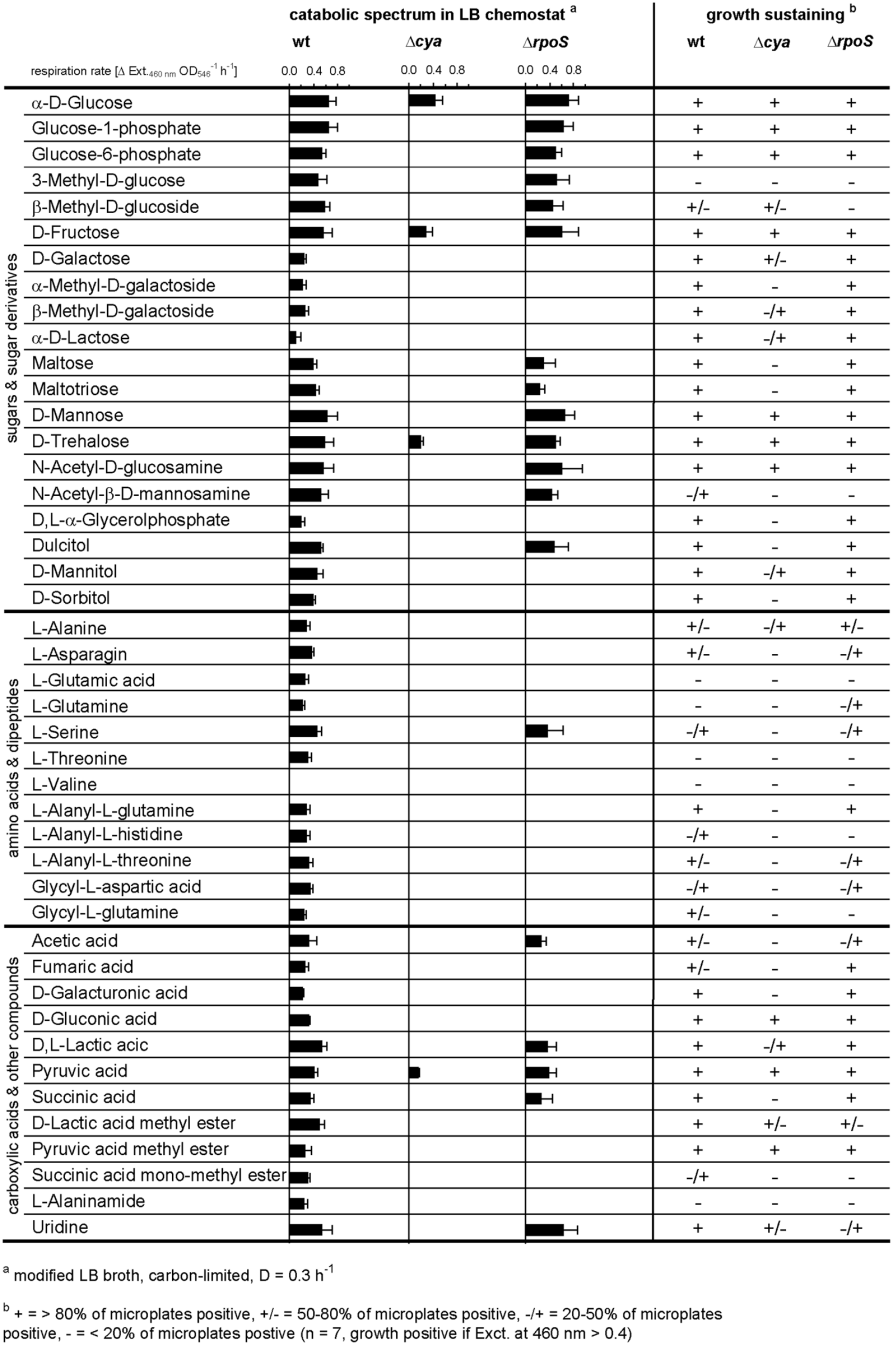
Substrate respiration rates of wild-type, Δ*crp*, Δ*cya* and Δ*rpoS* strains of *E*. *coli* in carbon-limited LB chemostat cultures and substrates that supported growth in BIOLOG plates. Respiration rates were determined as described in legend to Fig 2. For growth, all substrates leading to significant colour formation in any strain are shown. L-valine was included in the table as negative control.

**Fig 4:**
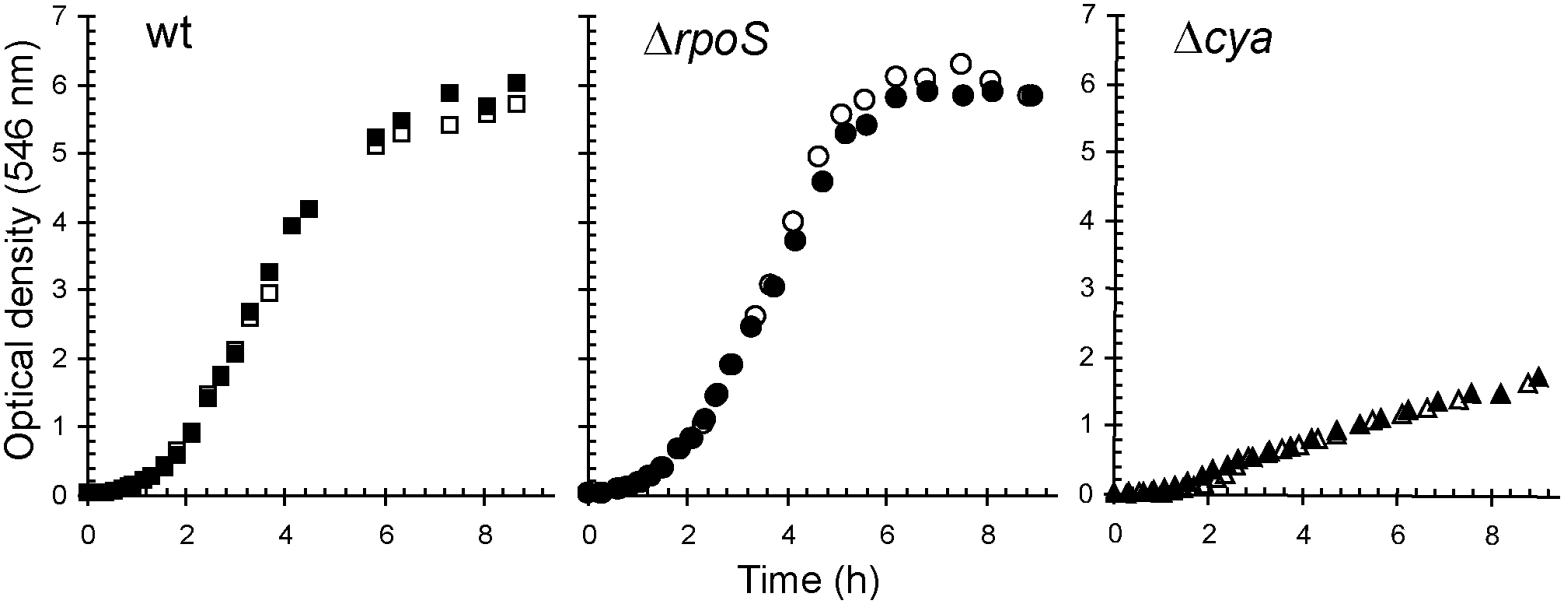
Growth of wild-type (■, □), Δ*rpoS* (●, ○), and Δ*cya* (▲, Δ) strains of *E*. *coli* in batch cultures with modified LB medium buffered at pH 7. Data from two individual experiments (black and white symbols).

In the wild-type strain strong induction of high-affinity binding proteins for e.g. maltose, ribose, galactose, dipeptides and glutamine occurred in response to carbon and energy limitation. This has repeatedly been observed in previous studies for both laboratory strains and natural isolates of *E*. *coli* [13,15,18]. In addition to the negative effect of the absence of cAMP on the expression of transport systems for alternative carbon substrates, many genes encoding enzymes of central catabolic pathways were down-regulated, e.g. components of the tricarboxylic acid cycle (TCA), the glyoxylate bypass, glycolysis, gluconeogenesis, the pentose phosphate pathway, the methylglyoxyl pathway, propionate metabolism and galactitol degradation (Table 2). Genes encoding subunits of ATP synthase were also negatively affected. Among the most down-regulated genes in the Δ*cya* strain were those for the galactitol degradation pathway (*gatD*, *gatZ* and *gatY*), propionate metabolism (*prpB*, *prpC* and *prpD*) and acetyl-CoA synthetase (*acs*). The genes belonging to the propionate metabolism are known to be cAMP-dependent, in addition σ^N^ is required for their transcription [59]. Acetyl-CoA synthetase (*acs*) was reported by others to be under the control of cAMP, Fnr and the flux of carbon through the acetate pathway [60,61]. Genes encoding enzymes of the tricarboxylic acid (TCA) cycle and glyoxylate bypass (AceA, AceB and GlcB) were also transcribed at a lower level in the absence of adenylate cyclase. A significant reduced activity of the glyoxylate bypass for Δ*cya* and Δ*crp* strains has been previously reported under glucose limitation [23,60,62]. The reduced expression of genes encoding components of the TCA cycle and the glycolysis pathway is in agreement with the significantly reduced _max,Glc_ of Δ*cya* and Δ*crp* strains in glucose-excess batch culture (Table 1). At the physiological level the mandatory presence of cAMP-CRP for induction of transport systems and catabolic pathways for alternative carbon substrates was reflected in a drastically reduced capability to respire and grow on carbon substrates other than glucose (Figs 2 and 3). The high catabolic flexibility of glucose-limited growing *E*. *coli* cultures reported previously [15] was abolished in strains without functional cAMP-CRP global regulation (Fig 2). The limited catabolic flexibility of these strains in glucose-limited chemostats resembled the pattern observed for wild-type *E*. *coli* cultivated under conditions of excess glucose [15]. Similarly, the expression of an enormous number of alternative catabolic pathways in wild-type *E*. *coli* observed in complex medium under carbon- and energy-limited conditions was completely abolished in the absence of functional adenylate cyclase (Fig 3). With the exception of L-serine, which was not oxidized by Δ*cya* cells, the BIOLOG respiration pattern of the Δ*cya* strain cultivated in a carbon-limited LB chemostat was comparable to that of fast-growing cells of the wild-type strain in LB batch cultures [15]. In agreement with the missing expression of catabolic pathways for alternative organic substrates, the growth yield of the Δ*cya* strain in modified LB medium was strongly reduced, both in batch and chemostat culture (Fig 4 and Table 3). By contrast, the maximal specific growth rate in the same medium (determined at low cell densities) was only slightly reduced in cAMP-CRP knock-out strains (Table 3), again suggesting that the expression of biosynthetic pathways is not influenced by this global regulatory mechanism.

**Table 3:** Effect of global regulatory mutations on growth kinetic parameters and specific hydroperoxidase activity of *E*. *coli* MG1655 in modified LB complex medium cultures at 37°C. Growth yield and specific hydroperoxidase activities were determined in carbon-limited LB chemostat cultures operated at a dilution rate of 0.3 h^-1^.

strain	μ_max_	Yield	HPI+II ^b^	HPII ^c^
	[h^-1^]	[g _dr. wt._ g _LB_^-1^]^a^	[μmol H_2_O_2_ min^-1^ mg_protein_^-1^]	
MG1655 (wt)	2.13	0.111	104	48.0
	(± 0.11)	(± 0.03)	(± 11)	(± 6.8)
Δ*rpoS*	2.04	0.092	50.7	0.7
	(± 0.04)	(± 0.011)	(± 13.0)	(± 0.3)
Δ*cya*	1.64	0.015	66.5	57.5
	(± 0.08)	(± 0.001)	(± 11)	(± 22)

^a^ biomass yield per amount of tryptone and yeast extract (2:1) present in the feed medium of carbon-limited chemostat cultures

^b^ combined activities of heat labile hydroperoxidase I (KatG) and heat stable hydroperoxidase II (KatE)

^c^ heat stable hydroperoxidase II activity (KatE)

An interesting exception to the general rule of down-regulation of transport systems in the Δ*cya* strain are metal transport systems, which were found to be up-regulated: *cusB* (involved in copper transport), *feoA* (involved in ferrous ion transport) and *fepE* (involved in ferric enterobactin transport) transcript levels were all 3-5-fold enhanced in the mutant (Table 2).

### RpoS is not required for the physiological response (hunger-response) of *E*. *coli* to carbon and energy limitation

Similarly to cAMP, the levels of the alternative sigma factor RpoS increase with decreasing specific growth rate in carbon- and energy-limited chemostat cultures and during the transition to stationary phase in batch culture [22,25]. This is the reason why RpoS was also called starvation or stationary phase sigma factor [63].

We compared the transcriptome of a Δ*rpoS* mutant to that of the wild-type strain under identical growth conditions in glucose-limited chemostat culture at a D of 0.3 h^-1^ (doubling time: 2.3 h). Genes with significant differential expression ratio are listed in Table 2 and S1 Table (cut-off: 3-fold difference of expression signal). The number of affected genes was lower than in the case of cAMP, 100 genes were found to be affected by the absence of RpoS, of which 52 were down-regulated and 48 up-regulated. Among the genes down-regulated many (42 of 52) were reported to be RpoS-dependent in previous transcriptome studies carried out in batch culture [32,33,35–37]. However, only one out of the 48 up-regulated genes had been reported to be up-regulated in the absence of RpoS in an earlier transcriptome study under batch stationary phase conditions [36]. Interestingly, the major part of genes detected at higher transcription levels in the Δ*rpoS* strain had a low expression ratio (between 3 and 5), which may be explained by the loss of competition between σ^D^ and σ^S^, resulting in an enhanced transcription of σ^D^-dependent genes. RpoS competes with RpoD for free RNA-polymerases and the loss of RpoS has an impact on gene expression of genes transcribed by σ^D^ due to the fact that more RNA-polymerases are bound with σ^D^ [34,64–66]. Only few transport systems were more than 3-fold up-regulated in the Δ*rpoS* strain, e.g., the allose uptake system (*alsB*) and the idonate/gluconate uptake system (*idnT*). Genes encoding components of transport systems were generally not down-regulated in the Δ*rpoS* strain, with the notable exceptions of *xasA*, which encodes an acid sensitive transport protein, *hdeD*, which has been suggested to be involved in acid stress resistance [67] and genes encoding ABC-type transporters of unknown specifity (*ydcS*, *ydcU*, *ego and yohC*). Some hypothetical genes (13) that were highly down-regulated in the Δ*rpoS* strain were also affected by cAMP, suggesting a co-regulation of the two global regulators.

Lack of RpoS reduced the transcription of only few metabolic genes, these are involved in propionate metabolism, the pentose phosphate pathway, glycolysis and biotin synthesis (Table 2). Surprisingly, the entire propionate operon (*prpBCDE*) was strongly down-regulated in the Δ*rpoS* strain, which confirms a previous transcriptome study [33]. In contrast to the Δ*cya* strain where genes encoding components of the TCA cycle were generally down-regulated, aconitate hydrase B (*acnB*), fumarate reductase (*frdB*) and succinate dehydrogenase (*sdhD*) were up-regulated in the Δ*rpoS* strain, which is in agreement with the reported down-regulation of *sdhD* at high RpoS levels [68].

In general, the Δ*rpoS* mutation had no negative effect on the transcription of the majority of transport systems and catabolic enzymes under glucose-limited growth conditions, excluding RpoS as important global regulator for the adaptation of *E*. *coli* to a restricted supply of carbon and energy sources.

The transciptome data recorded for the Δ*rpoS* strain matched results of the physiological characterization. The slight up-regulation of genes involved in the citric acid cycle by the loss of RpoS was in agreement with the slightly improved _max,Glc_ in glucose-excess batch cultures (Table 1). The limited effect of the absence of RpoS on the transcription of catabolic genes for alternative carbon substrates coincided with the results from BIOLOG respiration assays for glucose-limited growing Δ*rpoS* cells, which were to a large extent similar to wild-type cells grown under the same conditions (Figs 2 and 3). Exceptions were trehalose, mannitol, sorbitol and D-malate, which were oxidized at high rates by wild-type cells, but were either not oxidized or utilized only at reduced rates by the Δ*rpoS* mutant (Fig 2). The number of catabolic pathways expressed by the Δ*rpoS* strain in carbon-limited chemostat cultures with modified LB medium was considerably larger than that found in Δ*cya* cultures, although the capacity to oxidise many substrates was negatively affected, e.g., for D-sorbitol, L-alanine, L-alanyl-L-glutamine, gluconate and fumarate (Fig 3). For the Δ*rpoS* strain the spectrum of growth-sustaining organic substrates assessed on BIOLOG plates was almost identical to that of the wild-type strain (Fig 3), indicating that most uptake systems and catabolic enzymes can be expressed properly in the absence of functional RpoS.

Contrary to the observed negative effect of the Δ*cya* mutation on the expression of high-affinity binding proteins in response to carbon and energy limitation, loss of functional RpoS had no such effect (Fig 1). Also in contrast to the Δ*cya* and Δ*crp* mutants, the RpoS knock-out strain exhibited a similar or even increased fitness under carbon-limited growth conditions compared to the wild-type strain. Maximal specific growth rate (μ_max_), affinity for glucose (K_s,Glc_) and growth yields of the Δ*rpoS* strain were either similar or enhanced, both when growing in glucose mineral medium and in complex medium (Table 1 and Table 3, Fig 4). The observed improved affinity for glucose is in agreement with previous reports on positive effects of *rpoS* mutations on high-affinity transport of glucose in glucose-limited chemostat cultures [31,69,70].

### Effect of cAMP and RpoS on expression of stress defence genes under carbon- and energy-limited conditions

RpoS is known to play a crucial role in the response of *E*. *coli* to adverse physico-chemical conditions such as acidic pH, high osmolarity, changes in temperature and more, offering an explanation for the increased stress tolerance of stationary phase cells [63]. Here we discuss the observed differences in the transcriptomes between wild-type *E*. *coli* K12 MG1655, and RpoS- and cAMP/CRP-deficient isogenic mutants under balanced (steady-state) growth conditions (glucose-limited continuous cultures), and compare the results to those reported in the literature.

Surprisingly, only few stress defence genes (6) were found to be significantly negatively affected in cells of the Δ*rpoS* mutant strain during slow growth in glucose-limited chemostat culture (Table in S1 Table). Genes involved in acid resistance were affected, e.g. *gadB* encoding the glutamate decarboxylase isozyme and two periplasmic proteins located in the same operon (*hdeA* and *hdeB*). This down-regulation of genes involved in acid resistance is in agreement with transcriptome data obtained with a Δ*rpoS* strain in batch culture [33,36,37]. Two additional genes down-regulated in the Δ*rpoS* strain in our experiments are known to belong to the σ^S^-regulon (OsmE, a stress inducible lipoprotein, and Bfr, a bacterioferrin). However, both genes were also down-regulated in the absence of cAMP, although the effect of cAMP on *bfr* expression was less pronounced than that of RpoS (Table in S1 Table). Furthermore, the RpoS-dependent gene *marC*, involved in antibiotic resistance, was highly down-regulated and not affected significantly by cAMP. In contrast, genes necessary for resistance against antibiotics (*fsr* and *creD*) and phages (*dcrB* and *pspE*) were affected by both cAMP and RpoS.

Cyclic AMP was observed to regulate transcription of cold shock proteins in different ways. While mRNA levels of CspD and CspC were reduced in the Δ*cya* strain, transcription of CspF was up-regulated; this result is in agreement with a previous report [38]. Also one enzyme necessary for DNA repair (*umuC*) was found to be up-regulated in the absence of cAMP, which is in agreement with a previous study [39].

Unexpectedly, genes encoding two universal stress proteins, UspA and UspB, were down-regulated in the Δ*cya* strain but not in the Δ*rpoS* strain, suggesting the existence of a stress regulon under the control of cAMP-CRP. The expression of *uspB* was previously reported to be under the RpoS-regulon in some studies [33,37], while others could not find any difference in the expression of this gene under similar conditions [32,35,36]. These results challenge the assumed dominant role of RpoS for the survival of *E*. *coli* under different stress conditions and suggest that also the cAMP-CRP regulon might contribute significantly to stress resistance. Nevertheless, RpoS might influence the cellular levels of UspA and UspB by controlling the expression of other genes that control translation, folding and stability of these stress-defence proteins.

Activity of heat-stable hydroperoxidase II, encoded by *katE*, was suggested to be an indicator for RpoS-dependent gene expression [25,69]. Although the negative effect of the Δ*rpoS* mutation on hydroperoxidase II activity was again observed in our study in carbon- and energy-limited chemostat cultures (in both glucose mineral and LB medium, Table 1), DNA microarray data gave no indication of significantly decreased transcription of *kat*E (Table 3). The lacking negative effect of the *rpoS* knock-out mutation on the transcriptional level is in agreement with the results of other studies [32,33,35,36]. Possibly, RpoS exerts an indirect effect on the stability on KatE activity, e.g. by controlling the expression of genes that affect either translation, folding, activity or half-life of KatE. Expression of this gene was also not regulated by cAMP. Neither the transcription level of *katE* nor hydroperoxidase II activity was affected in the Δ*cya* and Δ*crp* strains (Table 1 and Table 3).

### Effect of cAMP and RpoS on expression of biosynthetic genes under carbon- and energy-limited conditions

The effect of either Δ*cya* or Δ*rpoS* global regulatory mutations on the expression of biosynthetic pathways was limited; only few of these genes were down-regulated in the absence of one or the other of the two global regulators (Table in S1 Table).

An exception was the gene *rpsV*, coding for a stationary-phase ribosome-associated protein that might control protein synthesis and prevent mistranslation occurring in stationary phase [71]. This gene was activated in an RpoS-dependent manner in glucose-limited continuous culture, whereas the absence of cAMP led to a strong down-regulation (Table in S1 Table).

Transcription of a gene encoding another ribosomal protein, RpmJ, was also down-regulated in the Δ*cya* strain; *rpmJ* belongs to the spc operon encoding 11 ribosomal proteins [72]. Furthermore, in the absence of cAMP mRNA levels of SecY were also reduced; this protein is a component of the type II secretory pathway for proteins [73] and plays an important role in the secretion of binding proteins to the periplasm [74]. This result is consistent with the observed reduced expression of most genes encoding periplasmic binding proteins in the absence of cAMP (Fig 1, Table 2); presumably expression and secretion are tightly coordinated.

Furthermore, cAMP seems to be involved in the regulation of the biosynthesis of histidine, as both *hisG* and *hisC* were down-regulated in the Δ*cya* strain.

A heterogeneous pattern was observed for transcription of genes encoding enzymes involved in nucleotide biosynthesis. Whereas several genes were transcribed at higher levels in both mutants (Table in S1 Table), transcription of 2-deoxyribose-5-phosphate aldolase (DeoC) was down-regulated in the absence of cAMP. *deoC* is member of the *deoCABD* operon, which is predicted to be regulated by cAMP [53]. Interestingly, *deoD*, a member of this operon, was reported in other studies to be not only cAMP—but also RpoS-dependent [35,38].

### Effect of cAMP and RpoS knock-out mutations on transcription of regulatory proteins

Global regulators often act via regulatory cascades by affecting the expression of other regulatory proteins that are specific for particular operons. In this sense, when grown in glucose-limited chemostat culture the two *E*. *coli* strains studied, carrying either a Δ*cya* or a Δ*rpoS* mutation, were affected in the transcription of 31 regulatory genes (Table in S1 Table). In this group of genes, the majority of those affected more than 3-fold were down-regulated in the absence of cAMP (23 out of 26), whereas 9 out of 13 were up-regulated when cells were lacking RpoS.

In the Δ*cya* strain, the genes down-regulated code for regulators controlling the transcription of operons and genes with functions ascribed to metabolic pathways, cell structures, DNA structure, DNA modification, sigma factors, sensor proteins, stress regulation and DNA-binding proteins. Only 4 such genes were down-regulated in the Δ*rpoS* strain and they are considered to be responsible for the regulation of functions assigned to cell structure and stress responses. Genes found to be up-regulated (9) are known to be involved in the regulation of proteins used in different functions, such as RNA modification, the *glc* operon for sugar transport, cell structure and sensory proteins.

Transcription of the four genes down-regulated in the Δ*rpoS* strain was also affected in the strain lacking cAMP; whereas *bolA* and *csrA* were down-regulated to comparable levels, *yiaG* and *dps* were considerably more affected by the lack of RpoS. BolA, the transcription of which is reported to be under the control of σ^S^ [75], regulates the morphology of cells [76] and seems to be involved in biofilm formation [77]. CsrA is a regulator of carbohydrate metabolism and affects glycogen degradation [78], glycogen biosynthesis, gluconeogenesis, and glycolysis [79,80]. CsrA has also a regulatory role in biofilm formation [81], and also plays a role in motility via post-transcriptional activation of the expression of the *flhDC* flagellar regulatory genes [82]. YiaG is a putative transcriptional regulator and was also found to be down-regulated in an Δ*rpoS* mutant in batch culture [35–37].

Interestingly, transcription of the *dps* gene was reduced in both mutant strains. Dps is an abundant protein in stationary phase cells and is involved in stress response [83], its main function is assumed to be the chelation of intracellular iron in order to prevent DNA damage [84]. Dps expression was reported to be regulated by RpoS and repressed by cAMP-CRP [85], which is in contrast to our results. The repression observed earlier in a Δ*crp* strain was suggested to occur indirectly and it was speculated that this de-repression was caused by an up-regulation of RpoS [86]. In our experiments we did not observe an up-regulation of RpoS in the Δ*cya* mutant and, therefore, the mechanism proposed by Joeng and colleagues [86] is unlikely. We assume that either other regulatory proteins or additional factors are involved in the regulation of *dps* as recently proposed [87,88].

Surprisingly, the lack of cAMP affected transcription of one sigma factor, RpoE, which encodes a sigma factor involved in the response to heat shock and envelope stress [89], and its negative regulatory protein (*rseA*). Activity of RpoE is increased upon the entry into the stationary phase and it has been reported to be negatively regulated by RseA and positively by ppGpp [90]. The down-regulation of RpoE strongly suggests that in the absence of cAMP the strain might not survive when exposed to different stresses such as heat shock and envelope stress. This observation might be related to the fact that the combined volume of the outer membrane and the periplasm of strains lacking CRP is larger than that of wild-type strains [91].

Regulatory proteins of several catabolic operons were also down-regulated in the Δ*cya* strain, e.g., SfsA controlling genes for maltose utilization, the repressor for galactitol utilization (GatR), and YehH which regulates molybdate metabolism. Transcription of genes encoding DNA-binding proteins H-NS and HU was also reduced. Interestingly, the mRNA levels of the *soxS* gene, coding for a regulatory protein involved in the response to oxidative stress, were found to be up-regulated in both mutants.

### Effect of cAMP and RpoS knock-out mutations on other cellular functions

In addition to the observed effects of the absence of cAMP and RpoS on catabolic functions and stress defence, few other cellular functions were affected in cells growing in glucose-limited chemostat culture at a dilution rate corresponding to half μ_max_.

In the Δ*cya* strain a variety of genes necessary for the synthesis of structural elements were down-regulated, e.g. genes coding for lipoproteins (*ybjP*, *lpp*, *nlpD* and *blc*), flagellum (*flgD*, belonging to class II), membrane biosynthesis enzymes (*ddpX*, *wbbL*, *yfcX* and *pssA*) and a putative sporulation protein (*ycgB*). Important is the finding that the major lipoprotein Lpp was down-regulated in the Δ*cya* strain, this lipoprotein is necessary for the stabilization and integrity of the bacterial cell envelope [92]. These unexpected and so far unknown results strongly suggest that in the absence of cAMP the cellular envelope is affected by the down-regulation of many genes and, hence, enzymes involved in the synthesis of membrane components, resulting in a weak incorporation of transporters and a, consequently, a poor functioning of the cell membrane.

In the absence of cAMP some genes were up-regulated; these genes are involved in membrane biosynthesis (*sfa*), one putative lipoprotein (*yehR*) and fimbrial proteins (*ycbQ* and genes encoded by the *fimAICDFGH* operon). The up-regulation of genes involved in motility in the absence of the cAMP-CRP complex was also reported in other studies performed with *E*. *coli* [38] and *V*. *cholerae* [58]. Interestingly, *ycbQ* and members of the *fimAICDFGH* operon were also up-regulated in the Δ*rpoS* strain, which is in agreement with a previous observation [33]. The latter operon encodes fimbriae of type 1 pili that are mannose-sensitive [93], these pili can bind to mannose residues to colonize surfaces.

Structural genes were less affected in the Δ*rpoS* strain. Genes for membrane biosynthesis (*kdsA*) and cell surface antigens (*uppS* and *rfe*) were up-regulated while only two genes were found to be down-regulated, *ycgB* and *blc*. The *ycgB* gene is also regulated by RpoS in *Salmonella enterica* serovar Typhimurium [94] and was reported to be under the control of RpoS in other transcriptome studies with *E*. *coli* [36,37]. Consistent with the down-regulation observed for the Δ*cya* strain in this study, the *ycgB* gene contains a predicted cAMP-CRP binding site in its promoter region [53]. In agreement with our transcriptome results for the Δ*rpoS* strain, transcription of the *blc* gene is induced at the beginning of the stationary phase in wild-type *E*. *coli* [95]. Its product might be involved in membrane repair, lipid storage or lipid transport [96].

Interestingly, genes involved in autoinducer 2 transport (*ego* or *lsrA*) and in biofilm production (*ycdS* and *ycdU*) were down-regulated both in the Δ*rpoS* and the Δ*cya* strains to similar levels, which indicates a regulatory cooperation of both global regulators as suggested by others [81,97].

## Conclusions

In our study, we have provided a comprehensive analysis of role of the global regulators cAMP and RpoS in *E*. *coli* under well-defined carbon- and energy-limited growth conditions. The lack of *rpoS* resulted in an enhanced fitness both under glucose-excess and glucose-limited conditions, whereas the lack of cAMP had a drastic negative effect on growth performance (Tables 1 and 3). The transcriptome data were confirmed by catabolome analysis, expression of periplasmic proteins and phenotypical analysis. The results obtained with the different methods were consistent. Thus, the picture obtained strongly suggests that the expression of a broad range of high-affinity uptake systems and of enzymes involved in the central metabolism (citric acid cycle and of glycolysis) were severely repressed in the absence of cAMP. Furthermore, the presented results are in agreement with flux analysis [62] and a transcriptome study performed in complex medium [38,39]. Hence, one can predict that the Δ*cya* strain must exhibit a worse affinity than the wild-type strain not only for glucose but also for a broad range of other carbon sources. On the contrary, absence or down-regulation of RpoS would result in increased competitiveness of *E*. *coli* in the environment. These results are in agreement with the trade-off between self-preservation and nutritional competence (SPANC [98]). High variability in RpoS expression from several *E*. *coli* strains was reported, where high RpoS expression resulted in high resistance at the cost of lower competitiveness, e.g., reduced catabolic flexibility [98,99].

The alarmone ppGpp, which is involved in the expression/stability of RpoS [100,101] and is necessary for the transcription of several genes belonging to the RpoS-regulon [102], has also been suggested to be involved in the SPANC balance [103]. The lacking negative effect of the *rpoS* knockout mutation on transcription of certain stress resistance genes might be due to direct regulation by ppGpp which is, like RpoS, present at high concentrations at low specific growth rates [104].

In this study we have demonstrated that the absence of cAMP affects also different classes of genes that are necessary for surviving and growing in the environment. Interestingly, several genes found to be regulated by RpoS were also affected in the Δ*cya* mutant, these genes belonged to all functional groups and numerous genes regulated by both global regulators in our experiments were previously reported to be under exclusive transcriptional control of RpoS. Our data confirm and extend recent suggestions that some genes are regulated by both regulators [38]. Weber and co-workers [37] have shown in a promoter analysis of Δ*rpoS* transcriptome data that about one half of all RpoS-regulated genes also possess a hypothetical cAMP-CRP binding motif in the promoter region. The transcriptome data reported here can serve as a useful starting point for further analysis of the involvement of cAMP in the regulation of different genes.

In summary we conclude that global regulation by cAMP-CRP, but not by RpoS is essential for growth and survival of *E*. *coli* in its natural habitats which also explains why *rpoS* but not *cya*/*crp* mutant strains can be isolated from the environment.

## Supporting Information

S1 Table *E*. *coli* genes whose expression levels were with significant up- or down-regulation in Δ*rpoS* and Δ*cya* strains (difference in average signal intensity compared to wild-type *E. coli* K12 ≥ 3 or ≤ -3, p-value ≤ 0.2) in glucose-limited continuous culture cultivated at D = 0.3 h^-1^. (DOCX)
