# Supplementary material for: Effect of Global Regulators RpoS and Cyclic-AMP/CRP on the Catabolome and Transcriptome of Escherichia coli K12 during Carbon- and Energy-Limited Growth
Source: PLoS One. 2015 Jul 23;10(7):e0133793. doi: 10.1371/journal.pone.0133793 (PMC4512719; doi:10.1371/journal.pone.0133793)
Supplement: S1 Table — (DOCX) [file pone.0133793.s001.docx]

**S1 Table.** *E. coli* genes whose expression levels were with significant up- or down-regulation in Δ*rpoS* and Δ*cya* strains (difference in average signal intensity compared to wild-type *E. coli* K12 ≥ 3 or ≤ -3, p-value ≤ 0.2) in glucose-limited continuous culture cultivated at D = 0.3 h^-1^.

| **Gene** | | **b no.** | | **Gene product** | | | **Ratio cAMP** | | | | **Ratio RpoS** | | | |  |  |
| --- | --- | --- | --- | --- | --- | --- | --- | --- | --- | --- | --- | --- | --- | --- | --- | --- |
| **Regulation** | | | |  | | |  | | | |  | | | |  |  |
| *sfsA*^f,g^ | | b0146 | | | probable regulator for maltose metabolism | | | 5.7 | | | |  | | | |  |
| *bolA*^e,h^ | | b0435 | | | possible regulator of murein genes | | | 7.5 | | | | 6.7 | | | |  |
| *hupB*^f,g^ | | b0440 | | | DNA-binding protein HU-beta, NS1 (HU-1) | | | 7.3^*^ | | | |  | | | |  |
| *ompT* | | b0565 | | | outer membrane protein 3b (a), protease VII | | | 6.5 | | | |  | | | |  |
| *dps*^d,e,h^ | | b0812 | | | global regulator, starvation conditions | | | 6.5 | | | | 19.9 | | | |  |
| *ybiJ* | | b0845 | | | putative DEOR-type transcriptional regulator | | | -5.9^**^ | | | | -4.9^**^ | | | |  |
| *clpA* | | b0882 | | | ATP-binding component of serine protease | | |  | | | | -3.0^**^ | | | |  |
| *wrbA* | | b1004 | | | trp repressor binding protein | | | 3.6 | | | |  | | | |  |
| *phoQ* | | b1129 | | | sensor protein PhoQ | | |  | | | | -3.3^**^ | | | |  |
| *minD* | | b1175 | | | cell division inhibitor, a membrane ATPase, activates minC | | | 4.1 | | | |  | | | |  |
| *Hns* | | b1237 | | | DNA-binding protein HLP-II (HU, BH2, HD, NS), pleiotropic regulator | | | 4.1 | | | |  | | | |  |
| *mokB* | | b1420 | | | regulatory protein, enables hokB expression | | | 4.4 | | | |  | | | |  |
| *ydcI* | | b1422 | | | putative transcriptional regulator LYSR-type | | | 5.6 | | | |  | | | |  |
| *ydeW* | | b1512 | | | putative transcriptional regulator, sorC family | | | 4.1 | | | |  | | | |  |
| *yneA* | | b1516 | | | putative LACI-type transcriptional regulator | | | 5.6 | | | |  | | | |  |
| *gatR_2*^c,i^ | | b2090 | | | split galactitol utilization operon repressor, fragment 2 | | | 13.0 | | | |  | | | |  |
| *yehH* | | b2116 | | | molybdate metabolism regulator, second fragment 2 | | | 7.9 | | | |  | | | |  |
| *rseA* | | b2572 | | | sigma-E factor, negative regulatory protein | | | 7.9 | | | |  | | | |  |
| *rpoE*^i^ | | b2573 | | | RNA polymerase, sigma-E factor heat shock and oxidative stress | | | 5.0 | | | |  | | | |  |
| *yfjN* | | b2630 | | | putative cell division protein | | | 3.8 | | | |  | | | |  |
| *csrA*^b^ | | b2696 | | | carbon storage regulator | | | 8.3 | | | | 7.8 | | | |  |
| *glcC*^b^ | | b2980 | | | transcriptional activator for glc operon | | |  | | | | -3.1 | | | |  |
| *damX* | | b3388 | | | putative membrane protein; interferes with cell division | | |  | | | | -3.5^**^ | | | |  |
| *yhiE*^e^ | | b3512 | | | putative regulator | | | 16.6 | | | |  | | | |  |
| *yiaG*^d,e,h^ | | b3555 | | | putative transcriptional regulator | | | 5.0 | | | | 34.7^*^ | | | |  |
| *soxS* | | b4062 | | | regulation of superoxide response regulon | | | -3.9^**^ | | | | -6.2^**^ | | | |  |
| *adiY* | | b4116 | | | putative ARAC-type regulatory protein | | | -5. 1^**^ | | | | -3.3 | | | |  |
| *yjjM*^i^ | | b4357 | | | putative transcriptional regulator | | | 6.8 | | | |  | | | |  |
| *bglJ* | | b4366 | | | 2-component transcriptional regulator | | |  | | | | -3.3^*^ | | | |  |
| *rsmC* | | b4371 | | | 16S RNA m2G1207 methylase | | |  | | | | -3.8^**^ | | | |  |
|  | | | | | | |  | | | |  | | | |  |  |
| **Cell structure** | | | | | | |  | | | |  | | | |  |  |
| *ybjP* | | b0865 | | | putative lipoprotein | | | 4.3 | | | |  | | | |  |
| *lolA* | | b0891 | | | periplasmic protein effects translocation of lipoproteins | | |  | | | | -3.6^**^ | | | |  |
| *ycbQ* | | b0938 | | | putative fimbrial-like protein | | | -6.4^**^ | | | | -3.7 | | | |  |
| *sfa*^i^ | | b0991 | | | suppresses fabA and ts growth mutation | | | -3.9^*^ | | | |  | | | |  |
| *ycdS* | | b1024 | | | putative outer membrane protein | | |  | | | | -3.0^**^ | | | |  |
| *flgD* | | b1075 | | | flagellar biosynthesis, initiation of hook assembly | | | 4.5 | | | |  | | | |  |
| *ycgB*^e,g,h^ | | b1188 | | | putative sporulation protein | | | 6.9 | | | | 11.9 | | | |  |
| *kdsA* | | b1215 | | | 2-dehydro-3-deoxyphosphooctulonate aldolase | | |  | | | | -3.3^**^ | | | |  |
| *ddpX* | | b1488 | | | D-Ala-D-Ala dipeptidase | | | 4.4 | | | |  | | | |  |
| *ydeS* | | b1504 | | | putative fimbrial-like protein | | | 5.5 | | | |  | | | |  |
| *lpp* | | b1677 | | | murein lipoprotein | | | 5.8 | | | |  | | | |  |
| *wbbL* | | b2031 | | | putaive lipopolysaccharide biosynthesis glycosyl transferase | | | 3.7 | | | |  | | | |  |
| **Continuation S1 Table** | | | | | | | | | | | | | | |  |  |
| **Gene** | **b no.** | | **Gene product** | | | | | | **Ratio cAMP** | | | | **Ratio RpoS** | | | |
| *yfcX* | b2341 | | bifunctional fatty acid oxidation complex protein | | | 5.3 | | | |  | | | |  |  |  |
| *pssA* | b2585 | | phosphatidylserine synthase, phospholipid synthesis | | | 5.1 | | | |  | | | |  |  |  |
| *rfe* | b3784 | | UDP-GlcNAc:undecaprenylphosphate GlcNAc-1-phosphate transferase | | |  | | | | -3.6^**^ | | | |  |  |  |
| *nlpD* | b2742 | | lipoprotein | | | 4.0 | | | |  | | | |  |  |  |
| *blc*^h^ | b4149 | | outer membrane lipoprotein (lipocalin) | | | 4.2 | | | | 5.5 | | | |  |  |  |
| *fimI* | b4315 | | fimbrial protein | | | -3.5^**^ | | | |  | | | |  |  |  |
| *fimD* | b4317 | | outer membrane protein export and assembly of type 1 fimbriae | | | -3.0^**^ | | | |  | | | |  |  |  |
| *fimG*^b^ | b4319 | | fimbrial morphology | | | -3.2^**^ | | | | -4.5^**^ | | | |  |  |  |
|  | | | | | | |  | | | |  | | | |  |  |
| **Stress response** | | | | | | |  | | | |  | | | |  |  |
| *fsr* | b0479 | | fosmidomycin resistance protein | | |  | | | | -3.6^**^ | | | |  |  |  |
| *cspD*^g^ | b0880 | | cold shock protein | | | 13.0 | | | |  | | | |  |  |  |
| *mdoG* | b1048 | | periplasmic glucans biosynthesis protein | | |  | | | | -3.2^**^ | | | |  |  |  |
| *umuC*^i^ | b1184 | | SOS mutagenesis and repair | | | -4.2^**^ | | | |  | | | |  |  |  |
| *pspE*^i^ | b1308 | | phage shock protein | | | 4.0 | | | |  | | | |  |  |  |
| *gadB*^b,e,f,h^ | b1493 | | glutamate decarboxylase isozyme | | |  | | | | 15.4 | | | |  |  |  |
| *marC* | b1529 | | multiple antibiotic resistance protein | | |  | | | | 10.0 | | | |  |  |  |
| *cspF* | b1558 | | cold shock protein | | | -5.7^*^ | | | |  | | | |  |  |  |
| *osmE*^d,h^ | b1739 | | activator of ntrL gene | | | 10.4 | | | | 10.2 | | | |  |  |  |
| *yeaA*^g^ | b1778 | | methionine sulfoxide reductase | | | 5.9 | | | |  | | | |  |  |  |
| *cspC* | b1823 | | cold shock protein | | | 4.2 | | | |  | | | |  |  |  |
| *cutC* | b1874 | | copper homeostasis protein | | | 4.5 | | | |  | | | |  |  |  |
| *bfr*^b,d,e,h^ | b3336 | | bacterioferrin, an iron storage homoprotein | | | 5.1 | | | | 14.3 | | | |  |  |  |
| *dcrB* | b3472 | | Resistant to lytic phage C1 | | |  | | | | -3.2^**^ | | | |  |  |  |
| *uspB* | b3494 | | universal stress protein B | | | 3.7 | | | |  | | | |  |  |  |
| *uspA* | b3495 | | universal stress protein A | | | 4.0 | | | |  | | | |  |  |  |
| *hdeB*^b,e^ | b3509 | | periplasmic protein | | |  | | | | 32.9^*^ | | | |  |  |  |
| *hdeA*^e^ | b3510 | | periplasmic chaperon of acid-denaturared proteins | | |  | | | | 23.0 | | | |  |  |  |
| *creD* | b4400 | | tolerance to colicin E2 | | | -3.6^**^ | | | |  | | | |  |  |  |
|  | | | | | | |  | | | |  | | | |  |  |
| **Protein processing** | | | | | | |  | | | |  | | | |  |  |
| *rpsV*^b,d,e,h^ | b1480 | | 30S ribosomal subunit protein S22 | | | 9.8 | | | | 17.0 | | | |  |  |  |
| *pheT* | b1713 | | phenylalanine tRNA synthetase, beta-subunit | | | 6.1 | | | |  | | | |  |  |  |
| *yfiA*^g^ | b2597 | | Stationary phase translation inhibitor | | | 6.7 | | | |  | | | |  |  |  |
| *rpmJ* | b3299 | | 50S ribosomal subunit protein L36 | | | 3.8 | | | |  | | | |  |  |  |
|  | | | | | | |  | | | |  | | | |  |  |
| **Amino acids biosynthesis** | | | | | | |  | | | |  | | | |  |  |
| *hisG* | b2019 | | ATP phosphoribosyltransferase | | | 4.2 | | | |  | | | |  |  |  |
| *hisC* | b2021 | | histidinol-phosphate aminotransferase | | | 4.6 | | | |  | | | |  |  |  |
| *aroC* | b2329 | | chorismate synthase | | | 7.9 | | | |  | | | |  |  |  |
| *ivbL*^f,g^ | b3672 | | ilvB operon leader peptide | | | 5.2 | | | |  | | | |  |  |  |
|  | | | | | | |  | | | |  | | | |  |  |
| **Nucleotide biosynthesis** | | | | | | |  | | | |  | | | |  |  |
| *apbA* | b0425 | | involved in thiamin biosynthesis, pyrimidine biosynthesis | | | -8.5^**^ | | | | -6.6^**^ | | | |  |  |  |
| *purM* | b2499 | | phosphoribosylaminoimidazole synthetase = AIR synthetase | | | -4.1^**^ | | | |  | | | |  |  |  |
| *purN* | b2500 | | phosphoribosylglycinamide formyltransferase 1 | | | -3.0^**^ | | | |  | | | |  |  |  |
| *deoC*^e,f,g^ | b4381 | | 2-deoxyribose-5-phosphate aldolase | | | -3.9 | | | |  | | | |  |  |  |
|  | | | | | | |  | | | |  | | | |  |  |
| **Miscellaneous functions** | | | | | | |  | | | |  | | | |  |  |
| *trs5_1* | b0259 | | IS5 transposase | | | 5.2 | | | |  | | | |  |  |  |
| *ycjK* | b1297 | | putative glutamine synthetase (EC 6.3.1.2) | | |  | | | | -3.4^*^ | | | |  |  |  |
| *ycjL* | b1298 | | probable amidotransferase subunit | | |  | | | | -4.0^*^ | | | |  |  |  |
| *rzpR* | b1362 | | putative Rac prophage endopeptidase | | | -4.5^**^ | | | | -4.5^**^ | | | |  |  |  |
| **Continuation S1 Table** | | | | | | | | | | | | | | |  |  |
| **Gene** | **b no.** | | **Gene product** | | | | | | **Ratio cAMP** | | | | **Ratio RpoS** | | | |
| *ynaK* | b1365 | | Rac prophage | | | -3.5^**^ | | | | -3.1^**^ | | | |  |  |  |
| *rsmC* | b4371 | | 16S RNA m2G1207 methylase | | | -4.1^**^ | | | | -3.8^**^ | | | |  |  |  |
|  | | | | | | |  | | | |  | | | |  |  |
| **Unknown or hypothetical functions** | | | | | | |  | | | |  | | | |  |  |
| *yaaA* | b0006 | | orf, hypothetical protein | | | | | |  | | | | 5.0 | | | |
| *yahO*^d,e^ | b0329 | | orf, hypothetical protein | | | | | | 14.7 | | | | 7.8 | | | |
| *ybaB* | b0471 | | orf, hypothetical protein | | | | | |  | | | | -5.3^**^ | | | |
| *ybaK* | b0481 | | orf, hypothetical protein | | | | | | -7.5^**^ | | | | -6.4^**^ | | | |
| *ybaS* | b0485 | | putative glutaminase | | | | | |  | | | | -3.7^*^ | | | |
| *ybeD* | b0631 | | orf, hypothetical protein | | | | | |  | | | | -3.0 | | | |
| *ybeL* | b0643 | | putative alpha helical protein | | | | | | 8.1 | | | |  | | | |
| *ybfB* | b0702 | | orf, hypothetical protein | | | | | | -22.3^**^ | | | | -24.5^**^ | | | |
| *ybgA*^d,h^ | b0707 | | orf, hypothetical protein | | | | | |  | | | | 8.0 | | | |
| *yccV* | b0966 | | orf, hypothetical protein | | | | | | 5.3 | | | | 4.8 | | | |
| *yccW* | b0967 | | putative methyltransferase | | | | | |  | | | | -3.1^**^ | | | |
| *yccM*^i^ | b0992 | | putative ferredoxin-type protein | | | | | | -3.2^**^ | | | |  | | | |
| *ycdP* | b1021 | | putative membrane protein | | | | | | -3.0^**^ | | | | 5.3 | | | |
| *ymdB* | b1045 | | putative polyprotein | | | | | | 5.2 | | | |  | | | |
| *grxB* | b1064 | | glutaredoxin 2 | | | | | | 5.4 | | | |  | | | |
| *ycfH*^e^ | b1100 | | putative hydrolase | | | | | | 6.0 | | | | 8.8 | | | |
| *ychH*^f,i^ | b1205 | | orf, hypothetical protein | | | | | | 6.8 | | | |  | | | |
| *ymjA* | b1295 | | orf, hypothetical protein | | | | | |  | | | | -4.1^*^ | | | |
| *ydbD* | b1407 | | orf, hypothetical protein | | | | | | -4.9^**^ | | | | -3.8^**^ | | | |
| *ydcX* | b1445 | | orf, hypothetical protein | | | | | |  | | | | 5.4 | | | |
| *yncH* | b1455 | | orf, hypothetical protein | | | | | | -3.3^**^ | | | | -3.3^*^ | | | |
| *yneC* | b1518 | | orf, hypothetical protein | | | | | | 4.1 | | | |  | | | |
| *ynfA*^d^ | b1582 | | putative transmembrane protein | | | | | | 8.7 | | | | 11.3 | | | |
| *ydgA*^h^ | b1614 | | orf, hypothetical protein | | | | | |  | | | | 7.0 | | | |
| *ydiZ*^d,e^ | b1724 | | orf, hypothetical protein | | | | | | 5.4 | | | | 8.9 | | | |
| *yeaC*^g^ | b1777 | | orf, hypothetical protein | | | | | | 3.8 | | | |  | | | |
| *yeaG*^e,h^ | b1783 | | orf, hypothetical protein | | | | | | 13.1 | | | | 22.2 | | | |
| *yobF* | b1824 | | orf, hypothetical protein | | | | | | 5.2 | | | |  | | | |
| *yebV* | b1836 | | orf, hypothetical protein | | | | | |  | | | | 23.9 | | | |
| *yebW* | b1837 | | orf, hypothetical protein | | | | | |  | | | | 5.1 | | | |
| *yodD*^e^ | b1953 | | orf, hypothetical protein | | | | | | 4.0 | | | | 7.5 | | | |
| *yeeI*^i^ | b1976 | | orf, hypothetical protein | | | | | | 23.3^*^ | | | |  | | | |
| *wbbK* | b2032 | | putative glucose transferase | | | | | | 4.1 | | | |  | | | |
| *elaB*^e,h^ | b2266 | | orf, hypothetical protein | | | | | |  | | | | 7.7 | | | |
| *yfbN* | b2273 | | orf, hypothetical protein | | | | | |  | | | | -3.0^**^ | | | |
| *yfdY* | b2377 | | orf, hypothetical protein | | | | | | 5.0 | | | |  | | | |
| *ucpA*^i^ | b2426 | | putative oxidoreductase | | | | | | 3.6 | | | |  | | | |
| *yfiL*^h^ | b2602 | | orf, hypothetical protein | | | | | |  | | | | 6.0 | | | |
| *yfjO* | b2631 | | orf, hypothetical protein | | | | | | 4.1 | | | | 8.0 | | | |
| *ygaF*^d,g,h^ | b2660 | | orf, hypothetical protein | | | | | | 6.1 | | | | 5.0 | | | |
| *ygaM*^h^ | b2672 | | orf, hypothetical protein | | | | | | 4.3 | | | | 8.6 | | | |
| *ygaD* | b2700 | | orf, hypothetical protein | | | | | | 4.8 | | | |  | | | |
| *ygdH* | b2795 | | orf, hypothetical protein | | | | | | 4.1 | | | |  | | | |
| *ygfJ* | b2877 | | orf, hypothetical protein | | | | | | 32.6^*^ | | | | 10.0 | | | |
| *glcG* | b2977 | | orf, hypothetical protein | | | | | |  | | | | -5.1 | | | |
| *yqiB* | b3033 | | putative enzyme | | | | | | 5.3 | | | |  | | | |
| *yqjC*^d,e^ | b3097 | | orf, hypothetical protein | | | | | | 3.8 | | | | 6.9 | | | |
| *yhcO*^e^ | b3239 | | orf, hypothetical protein | | | | | |  | | | | 6.1 | | | |
| *smg* | b3284 | | orf, hypothetical protein | | | | | | 5.7 | | | |  | | | |
| **Continuation S1 Table** | | | | | | | | | | | | | | |  |  |
| **Gene** | **b no.** | | **Gene product** | | | | | | **Ratio cAMP** | | | | **Ratio RpoS** | | | |
| *yjbB* | b4020 | | putative alpha helix protein | | | | | | -3.6^**^ | | | |  | | | |
| *yjbA*^g,i^ | b4030 | | putative membrane protein | | | | | | -6.2^**^ | | | |  | | | |
| *yjcB*^i^ | b4060 | | orf, hypothetical protein | | | | | | -3.4^**^ | | | |  | | | |
| *yjcH*^f,i^ | b4068 | | orf, hypothetical protein | | | | | | 9.1 | | | |  | | | |
| *phnB*^h^ | b4107 | | orf, hypothetical protein | | | | | |  | | | | 6.8 | | | |
| *yjdK* | b4128 | | orf, hypothetical protein | | | | | | -3.2^**^ | | | |  | | | |
| *yjfN* | b4188 | | orf, hypothetical protein | | | | | | 10.7 | | | |  | | | |
| *yjfO* | b4189 | | orf, hypothetical protein | | | | | | 63.3^*^ | | | | -3.4 | | | |
| *ytfI* | b4215 | | orf, hypothetical protein | | | | | | -5.1^**^ | | | |  | | | |
| *ytfJ* | b4216 | | orf, hypothetical protein | | | | | | 9.0 | | | |  | | | |
| *yjgW* | b4274 | | orf, hypothetical protein | | | | | | -3.5^**^ | | | | -5.9^*^ | | | |

^*^ p-value < 0.1, ^**^ p-value < 0.05

References: ^a^[1]; ^b^[2]; ^c^[3]; ^d^[4]; ^e^[5]; ^f^[6]; ^g^[7]; ^h^[8]; ^i^[9]

**References**

1. Dong, T, Kirchhof, MG, Schellhorn, HE. RpoS regulation of gene expression during exponential growth of *Escherichia coli* K12. Mol Genet Genomics 2008; 279: 267-277.

2. Dong, T, Schellhorn, HE (2009). Control of RpoS in global gene expression of *Escherichia coli* in minimal media. Mol Genet Genomics 2009; 281: 19-33.

3. Gosset, G, Zhang, ZG, Nayyar, SN, Cuevas, WA, Saier, MH. Transcriptome analysis of Crp-dependent catabolite control of gene expression in *Escherichia coli*. J Bacteriol 2004; 186: 3516-3524.

4. Lacour, S, Landini, P. σ^S^-dependent gene expression at the onset of stationary phase in *Escherichia coli*: Function of σ^S^-dependent genes and identification of their promoter sequences. J Bacteriol 2004; 186: 7186-7195.

5. Patten, CL, Kirchhof, MG, Schertzberg, MR, Morton, RA, Schellhorn, HE. Microarray analysis of RpoS-mediated gene expression in *Escherichia coli* K-12. Mol Genet Genomics 2004; 272: 580-591.

6. Salgado, H, Peralta-Gil, M, Gama-Castro, S, Santos-Zavaleta, A, Muniz-Rascado, L, Garcia-Sotelo, JS, *et al.*, RegulonDB v8.0: omics data sets, evolutionary conservation, regulatory phrases, cross-validated gold standards and more. Nucleic Acids Res 2013; 41: D203-D213.

7. Tan, K, Moreno-Hagelsieb, G, Collado-Vides, J, Stormo, GD. A comparative genomics approach to prediction of new members of regulons. Genome Res 2001; 11: 566-584.

8. Weber, H, Polen, T, Heuveling, J, Wendisch, VF, Hengge, R. Genome-wide analysis of the general stress response network in *Escherichia coli*: σ^S^-dependent genes, promoters, and sigma factor selectivity. J Bacteriol 2005; 187: 1591-1603.

9. Khankal, R, Chin, JW, Gosh, D, Cirino PC. Transcriptional effects of CRP* expression in *Escherichia coli*. J Biol Eng 2009; 3:13
